# Energy assessment of BIPV system in code-compliant residential building in cooling-dominated climates

**DOI:** 10.1371/journal.pone.0318786

**Published:** 2025-03-13

**Authors:** Saleh H. Alyami, Noman Ashraf, Fahad M. Alyami, Ali Alhamami

**Affiliations:** 1 Civil Engineering Department, College of Engineering, Najran University, Najran, Saudi Arabia; 2 Department of Building Engineering, College of Architecture and Planning, Imam Abdulrahman Bin Faisal University, Dammam, Saudi Arabia; 3 Civil Engineering Department, College of Engineering, Najran University, Najran, Saudi Arabia; SRM-RI: SRM Institute of Science and Technology (Deemed to be University) Research Kattankulathur, INDIA

## Abstract

This study investigates the effects of climate and geographical location on the building integrated photovoltaics (BIPV). An existing residential building was simulated by using IES-VE software for five different climatic zones of Saudi Arabia, which was in accordance with ASHRAE Standard 169–2021 and Saudi Building Codes (SBC) 601/ 602. The results showed that the annual energy production of rooftop PV systems ranged from 49,810.29 kWh to 60,204.29 kWh, with cities such as Najran and Tabuk having higher energy production due to higher solar radiation and better performance of PV systems. The average annual global radiation ranged from 188.15 kWh/m^2^ to 212.52 kWh/m^2^, with cities such as Najran and Tabuk having the highest radiation levels. The study found that solar radiation, temperature, cloud cover and regional climate patterns significantly influence monthly energy generation, with cities closer to the equator experiencing higher solar radiation and longer daylight hours. The study also highlighted the importance of considering angular, spectral, temperature and low radiation losses, which range from 2.47% to 2.71%, 0.84% to 1.36% and 8% to 15.4%, respectively for the studies locations. This study would shed light on the impact of climate and location on the performance of PV systems and would therefore be of great interest to policy makers, energy planners and solar industry professionals to make informed decisions about the deployment of rooftop PV systems in different climate regions meet. Enabling a more sustainable energy strategy and a successful transition to a low-carbon future.

**Table:** 

Symbols	
A	Net surface area
$A_{cf}$	Conditioned floor area
$C_{l}$	Air latent heat factor
$C_{s}$	Air sensible heat factor
$C_{t}$	Air total heat factor
$ES_{c}$	Cooling energy saving
${\text{H}}_{\text{R}}$	Sensible heat transfer flow by radiation
$H_{ig,l}$	Latent cooling load from internal heat gains
$H_{ig,s}$	Sensible cooling load from internal heat gains
$H_{vi,l}$	Latent ventilation/infiltration load
$H_{vi,s}$	Sensible and latent heat loads by ventilation/infiltration
$H_{vi,t}$	Total ventilation/infiltration load
$N_{oc}$	Number of occupants
*Q*	Ventilation/infiltration air flow rate
$Q_{i}$	Air leakage rate
Δh	Air enthalpy difference
ΔT	Temperature difference
Δw	Air humidity ratio difference
ST_C_	Cooling setpoint
U	Overall heat transfer coefficient

## 1. Introduction

Saudi Arabia features a wide variety of climates, from mild coastal areas to scorching deserts. It is one of the largest energy consumers in the world and a key driver of the country’s energy needs is the construction industry. Buildings in this sector, which include residential, commercial and industrial buildings, account for a significant portion of the country’s energy consumption. A study conducted by the Saudi Ministry of Energy, Industry, and Mineral Resources indicates that the building sector in Saudi Arabia uses about 70% of the nation’s energy consumption [1,2]. The main cause of this high energy consumption is the extensive use of air conditioning, lighting and other electrical appliances in buildings. To mitigate this problem and reduce the construction industry’s energy consumption, renewable energy sources - particularly solar photovoltaic (PV) systems - are becoming increasingly popular. Solar PV systems can provide a clean and sustainable energy source for buildings by using photovoltaic panels to convert sunlight into electricity. The nation is pushing the use of photovoltaic (PV) systems and other renewable energy sources to meet its energy needs and lessen reliance on fossil fuels. PV systems have the potential to produce electricity and lower energy costs, especially for residential use, which is why they are growing in popularity in Saudi Arabia [3]. In line with its Vision 2030 plan and the United Nations Sustainable Development Goals (SDGs), the government is actively promoting the growth of the solar industry, recognizing its potential and working to diversify the economy away from oil dependence. Saudi Arabia has submitted a Nationally Determined Contribution (NDC) to the Paris Agreement, which calls for a 24.1% reduction in greenhouse gas emissions by 2030. This goal includes both national and international collaboration [4]. Buildings account for about a third of Saudi Arabia’s total energy consumption, making them a key area for improving performance and reducing electricity consumption. To address these issues, the following measures are currently being implemented: (a) Saudi Building Code (SBC): The latest version of the SBC sets minimum requirements for the energy efficiency of building envelopes, air conditioning and lighting. This code is expected to raise the standard of newly constructed areas [5]. (b) Saudi Energy Efficiency Center (SEEC): SEEC promotes the use of energy efficient technologies in a range of industries, including construction.

Numerous studies highlight the significant potential of solar PV systems to meet Saudi Arabia’s energy needs. For example, Asif (2019) stated that rooftop PV systems could meet over 16% of the energy needs of King Fahd University of Petroleum and Minerals [6]. Dehwah and Asif numerically found that rooftop PV systems in residential buildings can meet significant electricity needs and reduce cooling loads [7] and is economic viability [8]. Al-Ghamdi and Alshaibani (2021) emphasized the need for optimal integration of PV technology into residential buildings to maximize their benefits [12]. Hamzah & Go (2023) demonstrate that a BIPV system in Kuala Lumpur can generate 679.72 MW annually, significantly reducing CO_2_ emissions while maintaining architectural aesthetics [9]. Similarly, Restrepo-Herrera et al. (2023) emphasize the importance of strategic module placement and total installed capacity in optimizing BIPV performance in Colombia [10]. Fazelpour et al. (2018) analyze BIPV systems across three Iranian cities, finding that building orientation significantly affects energy generation, with south-facing installations yielding the highest efficiency [11]. Furthermore, Amini Toosi et al. (2022) explore the decarbonization potential of BIPV combined with thermal energy storage, achieving a 21.42% reduction in CO_2_ emissions over 30 years [12]. Collectively, these studies underscore BIPV’s role in advancing sustainable building practices in various climatic contexts. Al-Ghamdi and Alshaibani elaborated on the potential of solar energy in the residential real estate market and emphasized the importance of optimally integrating PV technology into buildings [13]. In his analysis of residential hybrid PV-diesel battery power systems, Shaahid et al. discovered that these systems could be a practical means of supplying energy to various provinces across the country [14]. Tsalikis and Martinopoulos numerically found that solar energy systems are capable of meeting at least 76% of the primary energy needs of residential buildings in Greece, with a payback period of less than seven years [15]. Iman et al. proposed PV system that can generate 87% of the electricity needs of an apartment in Saudi Arabia [16]. Several researchers have found that the payback period of solar PV systems can range from 5 to 7 years [17–19]. Rooftop photovoltaic (PV) systems and larger solar power plants may be used to reduce dependence on fossil fuels and reduce electricity consumption from the grid [20,21]. Al-Salem and others. (2017) studied the operation of a rooftop photovoltaic system in Riyadh and found that it accounted for 22.4% of the building’s total electricity consumption [22]. Awan stated that Tabuk is the most practical location for a photovoltaic system, especially in summer when demand is highest [23]. Almarshoud (2016) also reported high energy productivity for a number of sites across the country [24].

Review of previous work shows that most studies on rooftop photovoltaic (PV) systems have been conducted on educational or commercial/office buildings, while few studies have addressed the policy and regulatory frameworks supporting the installation of PV system. However, comparative analyzes that systematically evaluate the energy generation potential of these systems under different climatic conditions in cooling dominated climates are lacking. Furthermore, there is a notable gap in the literature regarding the influence of climatic variables - such as temperature, humidity and solar radiation - on energy production, as well as the effects of climatic locations on angular, spectral, temperature and small radiative losses from installed rooftop PV systems could significantly improve the design and implementation of BIPV systems tailored to specific environments. Accordingly, the current study numerically examines the net energy savings potential and the associated environmental impacts of photovoltaic systems by exposing it to five different climatic locations of KSA by Koppen [25], ASHRAE [26], and IECC (developed by ICC) [27].

## 2. Materials and methods

### 2.1. Simulation software and building specification

This study used the IES-VE (Integrated Environmental Solutions – Virtual Environment) software to estimate the energy consumption of a building and the performance of its photovoltaic (PV) system. IES-VE is a widely recognized building performance analysis tool widely used in the design and construction of net-zero energy buildings, with a focus on sustainability goals and optimization of HVAC systems [28].

Net-zero energy buildings (NZEBs) are structures designed to produce as much renewable energy as they consume annually. These buildings significantly reduce energy demand through efficiency measures and offset remaining demand with renewable technologies [29]. The software’s ability to model the thermal, optical and electrical properties of installed PV systems, as well as its integration with climate data, make it a valuable tool for evaluating the performance of integrated energy systems. The study demonstrates the suitability and reliability of IES-VE as a simulation tool for scientific purposes, with previous studies showing a 0–10% discrepancy between field measurements and IES-VE simulation results [30,31]. The study was conducted on a two-story residential building for five different climate zones of Saudi Arabia. The study building’s details and physical attributes are displayed in Table 1, and the thermal and optical characteristics of the PV modules taken into consideration for the analysis are displayed in Table 2. To optimize energy generation, the PV modules were positioned with a south-facing orientation and their tilt angle equal to the region’s latitude [32], which varied between 17 ° and 30 °. The set-point temperature, humidity, light power density (LPD), equipment power density (EPD), and number of occupants (occupancy) are among the ASHRAE (American Society of Heating, Refrigeration, and Air Conditioning Technicians) compliant input parameters in the numerical domain model.

**Table 1:** Building physical and operational characteristics.

Type of building	Residential building
No. of Floors	2 Floors
Orientation	North-South
External wall	200 mm CMU, 10 mm outside cement plaster, 20 mm inside gypsum plaster (U-Value = 2.17 W/m^2^ K)
Roof	150 mmm concrete slab with 10 mm built up roofing, 16 mm inside gypsum plaster (UValue = 2.4 W/m^2^ k)
Glazing	6 mm single clear glass (U-Value = 5.75 W/m^2^ K)
Floor Height	4.2 m
Longitude	50.17 ° E
HVAC system	All air system and VRV system
Set point temperature	20–24 °C
Occupancy	0.02 person/m^2^
Lighting power density	6 W/m2
WWR	1:6.5

**Table 2:** Characteristics of PV Module.

Specification	Value
Cell Type	Mono-crystalline
Cell Arrangement	144 [2 X (12 X 6)]
Efficiency	19.5%
Nominal Max. Power (Pmax)	430 W
Opt. Operating Voltage (Vmp)	40.3 V
Opt. Operating Current (Imp)	10.68 A
Operating Temperature	−40°C ~ + 85 °C
Max. Series Fuse Rating	20 A
Open Circuit Voltage (Voc)	48.3 V
Short Circuit Current (Isc)	11.37 A
Power Tolerance	0 ~ + 10 W
Temperature Coefficient (Pmax)	−0.35%/ °C
Temperature Coefficient (Voc)	−0.27%/ °C
Temperature Coefficient (Isc)	0.05%/ °C
Nominal Module Operating Temperature	42 ± 3°C

### 2.2. Weather data and climatic classification

**Fig 1:**
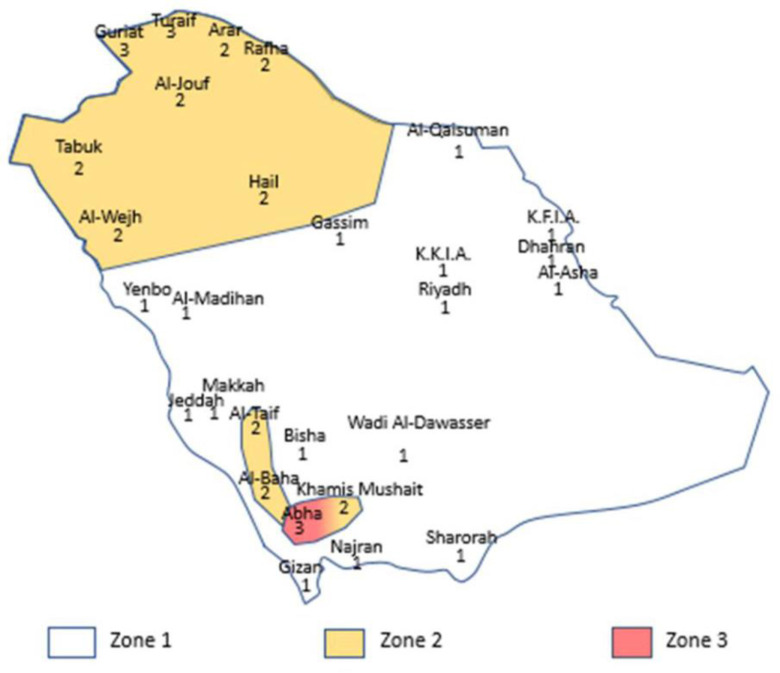
Map of Saudi Arabia, showing different cities and climate zones Saudi Energy Conservation Code: Buildings except Low-Rise (Residential) Buildings (SBC 601) [34].

The climatic classifications provided by Koppen [25], ASHRAE [26], and IECC (developed by ICC) [27] are globally recognized. Koppen has five (A to E) climate categories based on temperature and precipitation. In this classification, all cities of KSA except Abha are designated as ‘BWh’, which indicates tropical desert climate. Abha is denoted by ‘Cwa’, indicating humid subtropical climate. ASHRAE has eight thermal climatic zones (ranging from subarctic/artic to extremely hot), which are mainly defined by heating and cooling degree days (HDD and CDD). A similar classification method is followed by IECC. The Saudi Arabian cities are cooling-dominated, where the share of cooling load of buildings is significantly higher than the heating load [33]. Accordingly, the Saudi Energy Conservation Code (SBC 601-CR) Saudi Energy Conservation Code: Buildings except Low-Rise (Residential) Buildings (SBC 601) [34] has categorized the Saudi Arabian cities into three climatic zones based on CDD as extremely hot (Zone 1), very hot (Zone 2), and hot (Zone 3) as depicted in Fig 1. Table 3 consolidates the weather data of these cities, and Table 4 summarizes their local and global climatic classifications.

**Table 3:** Weather data of selected climatic locations of Saudi Arabia.

Sr. no.	Cities	Latitude	Longitude	DBT (Avg)	DBT (Min)	DBT (Max)	GHR (Avg)	Altitude	CDD	HDD	DBT (Max)
				^0^ C	^0^ C	^0^ C	Wh/m^2^	(m)	(Days)	(Days)	°C
1	Abha	18.23 N	42.65 E	18.7	9.8	28.1	494.8	2093	1240	507	34
2	Dammam	26.45 N	49.82 E	26.7	16.9	38.1	399.0	12	3668	283	49.2
3	Najran	17.62 N	44.42 E	25.8	12.8	36.3	426.5	1212	3222	226	42.7
4	Riyadh	24.70 N	46.73 E	26.8	15.9	37.9	405.9	768	3561	380	47.6
5	Tabuk	28.38 N	36.60 E	22.1	10.6	34.7	400.6	622	2592	650	44.2

*(DBT-Dry Bulb Temperature; GHR – Global Horizontal Radiation; Avg-Average; Min-Minimum; Max-Maximum)*

**Table 4:** Climatic classification of studied location as per ANSI/ASHRAE 169-2021 and Saudi Building Code (SBC) 601/602.

Sr. no.	Cities	ANSI/ASHRAE 169-2021 [26,35]		Saudi Building Code (SBC 601/602) Saudi Energy Conservation Code: Buildings except Low-Rise (Residential) Buildings (SBC 601) [34]		Koppen [25,36]	IECC Saudi Energy Conservation Code: Buildings except Low-Rise (Residential) Buildings (SBC 601) [34]
		Climate Classification	Zone	Climate Classification	Zone		
1	Abha	Warm Dry	3B	Hot	3	Cwa	3A & 3B
2	Dammam	Extremely Hot Dry	0B	Extremely Hot	1	BWh	1
3	Najran	Very Hot Dry	1B	Extremely Hot	1	BWh	1
4	Riyadh	Very Hot Dry	1B	Extremely Hot	1	BWh	1
5	Tabuk	Hot Dry	2B	Very Hot	2	BWh	2

### 2.3 Mathematical model

In this study, only cooling energy demand was estimated (except for the base case) because the study locations are generally cooling-dominated. The total annual cooling load was estimated as the sum of all sensible and latent heat gains. The mathematical equations employed for the simulation are listed here [37]:


$$H_{C}=U*A*CLTD\text{Watts}$$ (1)

$$H_{R}=A*SHGC*SC*CLF$$ (2)

$$H_{vi,s}=C_{s}Q\Delta T$$ (3)

$$H_{vi,l}=C_{l}Q\Delta w$$ (4)

$$H_{vi,t}=C_{t}Q\Delta h$$ (5)

$$\text{Air leakage rate,}Q_{i}=ACH\left(\frac{V}{3.6}\right)$$ (6)

$$H_{ig,s}=136+2.2A_{cf}+22N_{oc}$$ (7)

$$H_{ig,l}=20+0.22A_{cf}+12N_{oc}$$ (8)

The notations in equations (1) to (8) are defined in the nomenclature section and also provided in ref [31]. The annual saving in cooling energy (*CE*) consumption of the building was estimated as follows:


$$\text{Cooling energy saving}\left(ES_{c}\right)=\frac{CE_{n}-CE_{b}}{CE_{b}}\times 100\%$$ (9)

where subscript *n* denotes cases 1, 2 and 3, and *b* denotes base case.

## 3. Validation and calibration of simulation program

**Fig 2:**
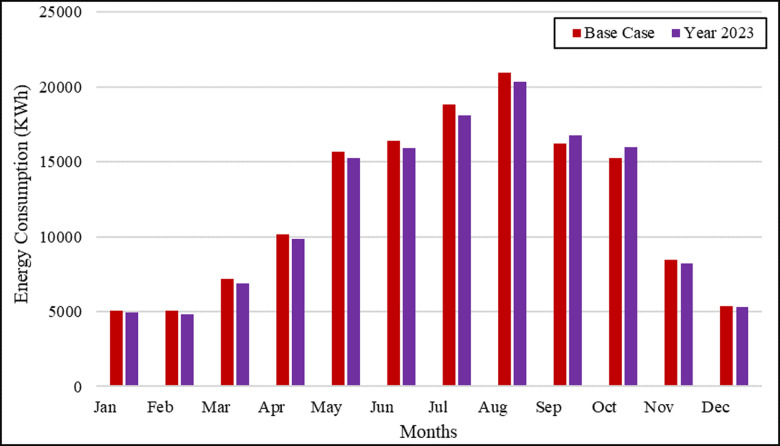
Comparison of simulated and actual electricity consumption (2023).

The validation and calibration of simulation results were performed by comparing the predicted monthly electricity consumption of a base case model with actual energy billing records from an existing residential building in Riyadh, KSA. The validation and calibration of the simulation results were carried out using monthly billing records of electricity consumption of an existing residential building in Riyadh, KSA. A base case model was developed using all collected information such as occupancy, lighting power density (LPD), equipment power density (EPD), and HVAC system conforming to the existing building’s ASHRAE standard [31] and calibrated based on this monthly energy consumption bill data for the year 2023. Fig 2 shows the monthly electricity consumption predicted by IES-VE for the building base model and the actual energy bill for 2023. It is worth noting that the difference in results between the simulation program and the electricity bill data is less than 1.75% lower than that of Iqbal and Al-Hamoud [38], who studied the energy conversion measures for office buildings in the same region of KSA, and therefore can be considered reasonably acceptable.

## 4. Results and discussion

In this section, the most important results of the work are summarized and discussed. The analysis of the thermal and energy performance of PV roof systems was carried out using the energy simulation software IES-VS for various climatic locations in Saudi Arabia.

### 4.1. Effect of climate and location on building’s energy consumptions

**Fig 3:**
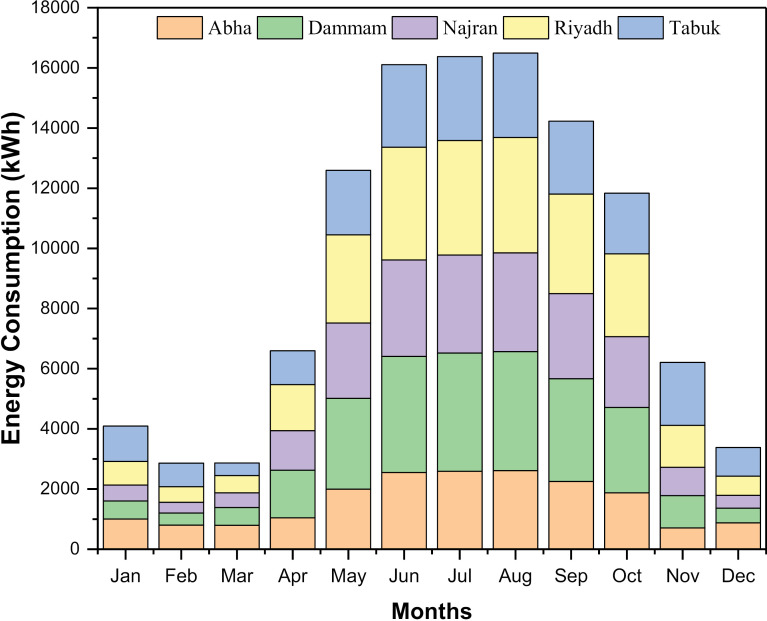
Monthly Building energy consumption for various locations.

**Fig 4:**
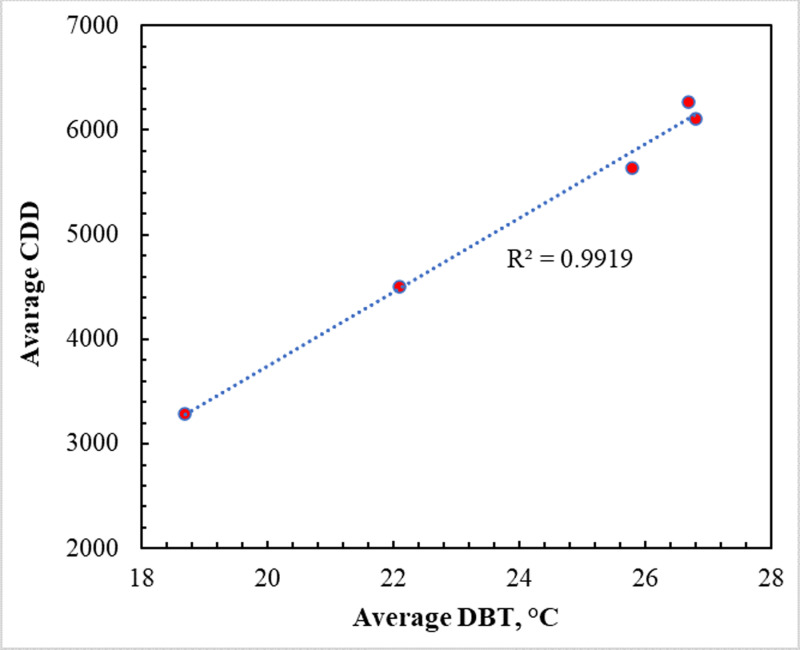
Correlation between DBT and CDD.

A residential building’s monthly energy consumption patterns for different climatic conditions are shown in Fig 3. The energy consumption decreases in winter (January, February, and December) in all cities owing to its fewer daylight hours, less sunlight, and possibly less need for cooling during these times [31,33]. On the other hand, all cities have higher energy consumption in the summer months of June, July, and August. This is because the higher temperatures at this time of year result in greater cooling needs. The cities studied exhibit differences in cooling degree days (CDD), regional energy demand, and climatic conditions, all of which contribute to annual building energy consumption [33]. Due to year-round hot and humid weather and scorching desert cities in the center (Riyadh) and coastal region (Dammam) recorded high energy consumption of about 25,000 kWh. Due to the relatively cool climate, hill towns like Abha have a moderate consumption of around 19,000 kWh. Nevertheless, the energy requirement is higher in the summer. The average cooling load in southern regions such as Najran is high at 21,500 kWh, suggesting a greater reliance on electrical appliances. Northern cities such as Tabuk have a high winter heating load in addition to summer cooling, resulting in an annual consumption of 21,500 kWh. The regression study of the correlation between different meteorological data, as shown in Fig 4, showed that DBT and CDD were the best fit with an R2 value of 0.9919. In general, energy demand shows a bimodal pattern depending on the climate zone, with peaks in summer and winter. In warm climates, energy consumption is predominantly determined by room cooling. In the cooler/transitional regions, usage is influenced by both heating and cooling [39]. The simulations provide insightful information about the use of locally specific efficiency solutions and environmentally friendly renewable energy sources.

### 4.2. Effect of climate and location on monthly and annual energy generation by the rooftop photovoltaics system

**Fig 5:**
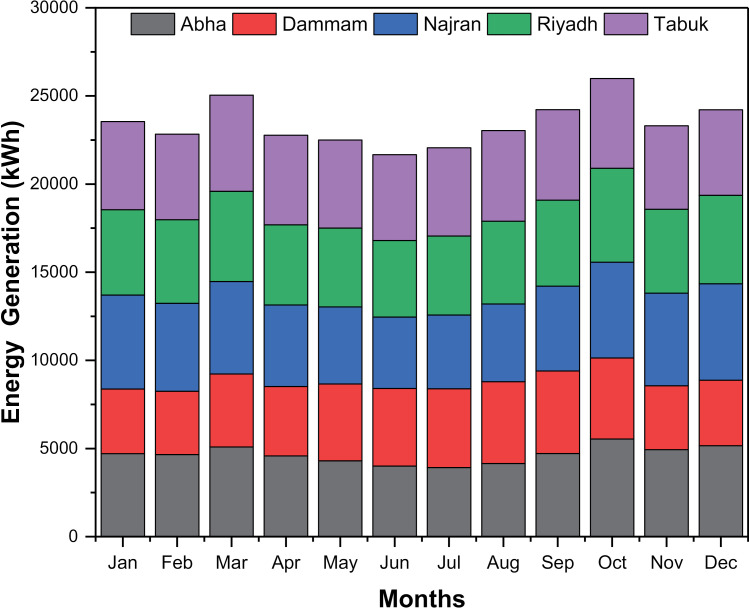
Monthly energy generation by rooftop PV system under various locations.

**Fig 6:**
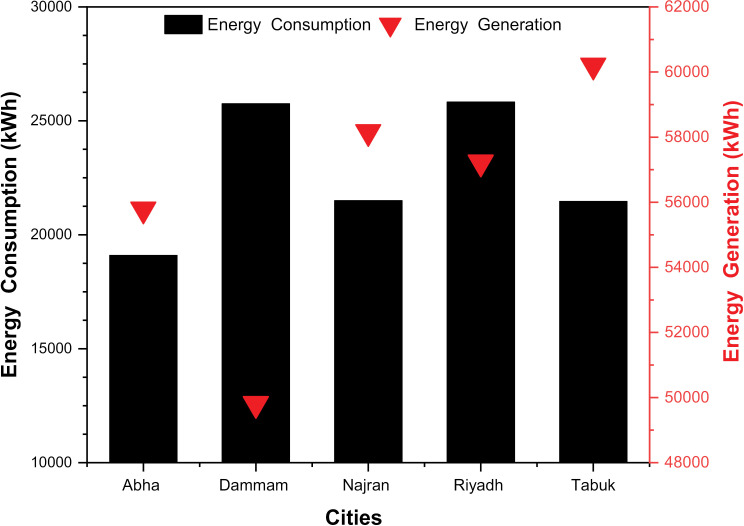
Annual energy consumption and generation for different locations.

The monthly energy production from rooftop PV systems at different climate locations is shown in Fig 5. An analysis of monthly energy production shows differences between different cities and months. These variations are influenced by factors such as solar radiation, temperature, cloud cover and regional climate characteristics [40]. In the months of January and February, energy production in all cities is relatively low compared to other months. This is due to shorter daylight hours, less sunlight, and unfavorable weather conditions during these winter months. Cities with higher energy production during this period include Najran and Tabuk, which may experience milder winters and better sunshine conditions [41]. From March to May, there is a general increase in energy production in all cities. This corresponds to the transition from winter to spring, with longer daylight hours and higher solar radiation. The cities of Najran and Tabuk continued to show higher energy production during this period, indicating their favorable solar resource potential and climate, which are more suitable for PV systems. In the summer months of June to August, energy production in most cities remains relatively stable or declines slightly. The high temperatures and increased load on air conditioning during these months can lead to higher energy consumption, negating the potential benefits of solar radiation. However, cities such as Dammam and Tabuk have comparatively higher energy production, suggesting that they are able to use solar energy efficiently despite the hot climate. Energy production tends to increase again from September to November as temperatures moderate and solar radiation increases. The cities of Najran and Tabuk consistently show higher energy production during this period, suggesting that they are suitable for PV systems all year round. In December, energy production decreased in most cities due to shorter daylight hours and less solar radiation. However, cities such as Najran and Tabuk still have relatively higher energy production compared to others, indicating that their solar energy potential remains relatively large even in the winter months. Fig 6 shows the annual energy consumption of buildings and the energy production of rooftop PV systems at different locations. The annual energy consumption in Abha is relatively low among the studied cities. The differences in energy consumption and energy production illustrate the influence of various climatic factors (temperature, humidity, solar radiation, and wind speed) that influence the energy requirements of buildings. In terms of energy production, cities such as Abha, Najran, and Tabuk, with rooftop PV systems that generate more energy than they consume, demonstrate the effectiveness of using solar energy and the potential for expanding rooftop PV systems. However, in cities such as Dammam and Riyadh, where energy production is slightly below consumption, further research may be needed to identify the factors limiting the full exploitation of solar energy potential. The variations in monthly energy production can be attributed to several climatic factors. Solar radiation is a main driver of energy production in PV systems, with higher radiation leading to higher energy yields [42]. In addition, temperature and cloud cover affect the performance of PV systems as they affect the efficiency and operation of solar panels [43,44]. Cities closer to the equator, such as Najran and Tabuk, generally experience higher levels of sunlight and longer daylight hours throughout the year. This contributes to their consistently higher energy production potential. In addition, regional climatic features, such as the presence of deserts or proximity to the coast, can influence the availability of solar resources and energy production [41,42].

### 4.3. Variation in annual energy generation with average global irradiation for different locations

**Fig 7:**
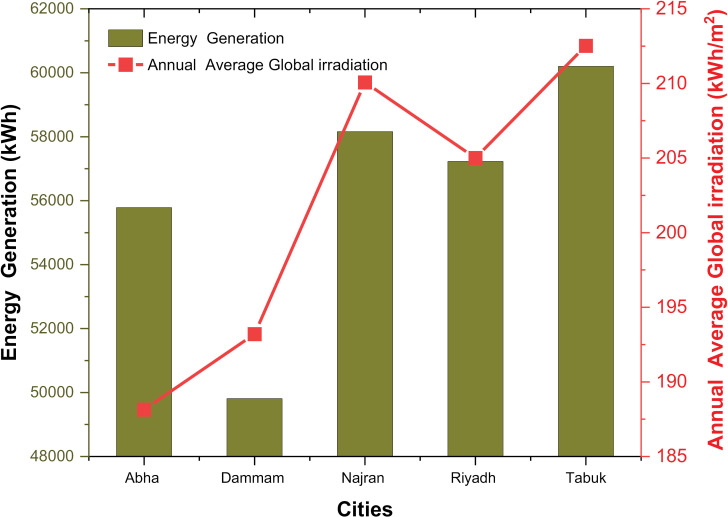
Annual energy production with average global radiation for different locations.

The annual energy production and average global irradiance for different locations of the rooftop PV systems are shown in Fig 7. The annual energy production from PV roof systems in the cities analyzed is between 49,810.29 kWh and 60,204.29 kWh. These figures represent the total electricity production of PV systems over the course of a year. The higher energy production observed in cities such as Najran and Tabuk indicates favorable solar conditions and the potential for greater energy production. These regions receive higher solar radiation, which has a positive effect on the performance of the PV system [45]. Other factors that influence energy production include system capacity, efficiency, shading, and maintenance practices [46,47]. It is important to note that energy production numbers should be evaluated in the context of the electricity needs of the buildings or facilities they serve. In order to ensure optimal performance and cover the energy needs of the respective locations, the correct dimensioning and design of the PV systems are crucial [41,48]. The annual average global irradiation received by rooftop PV systems is between 188.15 kWh/m2 and 212.52 kWh/m2. This metric represents the amount of incident solar energy per square meter of the PV system area. The higher average global irradiance observed in cities such as Najran and Tabuk suggests greater availability of solar resources in these regions. This can be attributed to factors such as latitude, climate, atmospheric conditions, and local terrain [49]. These cities have higher solar energy potential, which is reflected in increased energy production for rooftop PV systems [50,51]. The annual average global irradiation is a crucial parameter for the design and dimensioning of PV systems. It helps to determine the appropriate capacity of the system and estimate the energy production potential. In addition, it helps to assess the economic viability and return on investment of PV systems. The results show the connection between annual energy production and the annual average global irradiation in rooftop PV systems. Cities with higher average global irradiance tend to have higher energy production potential. The differences in energy production and average global radiation between cities can be attributed to geographical and climatic factors. Regions that are closer to the equator or have more favorable solar radiation generally receive higher solar radiation, resulting in higher energy production [52]. To improve the analysis, it is recommended to collect additional data on the specific PV system configurations, including system capacity, efficiency, and tilt angle. This would enable a more comprehensive assessment of the performance and energy production potential of rooftop PV systems.

### 4.4. *Variation in annual energy generation equivalent CO*_*2*_ *emissions*

**Fig 8:**
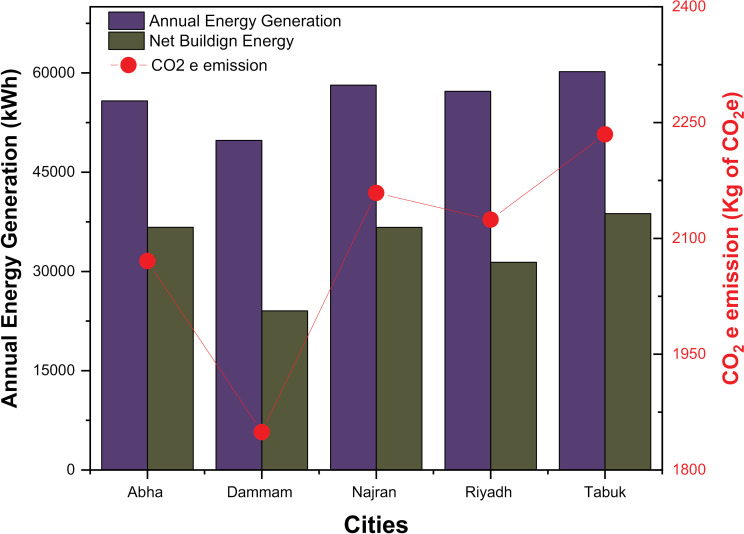
Annual energy production and equivalent CO_2_ emissions for different climate locations.

An important measure of rooftop PV systems’ environmental and sustainable benefits is the amount of energy they generate annually and the amount of CO_2_ they reduce. The performance and environmental impact of rooftop PV systems in various cities are demonstrated in Fig 8, which also displays the annual energy generation, net building energy, and equivalent reduction in CO_2_ emissions. The annual energy production from PV roof systems in the cities analyzed is between 49,810.29 kWh and 60,204.29 kWh. The higher energy production observed in cities such as Najran and Tabuk indicates favorable solar radiation conditions in these regions [51]. It is important to note that energy production is influenced by factors such as system capacity, orientation, shading, and the efficiency of the PV modules [44,46,50]. Net building energy refers to the total energy the building uses, after taking into account the energy generated by its rooftop PV system. The results show a net building energy of 24,058.19 kWh to 38,738.707 kWh. The lower net building energy observed in Dammam suggests that the PV system in this city contributes significantly to meeting the building’s energy needs. Conversely, cities such as Riyadh and Tabuk have higher net building energy, suggesting that the contribution of the PV system is comparatively lower. Further research is needed to understand the factors that affect the building’s net energy, such as: energy efficiency measures of the building, occupant behavior, and energy consumption patterns. The corresponding reduction in CO_2_ emissions represents the environmental advantage that the PV roof system achieves in terms of avoided greenhouse gas emissions. The results show CO_2_ emission reductions ranging from 1,848.95796 kg to 2,234.78324 kg. The higher CO_2_ emission reductions observed in cities such as Najran and Tabuk suggest that rooftop PV systems in these regions have a significantly more positive impact on reducing CO_2_ emissions. This is due to the higher energy production and relatively higher carbon intensity of local grid power. It is important to note that the calculation of CO_2_ emissions reductions assumes that the electricity generated by the PV system replaces grid electricity generated by fossil fuel-based power plants. Actual emissions reductions may vary depending on the specific network mix and its carbon intensity. The results show that rooftop PV systems in the cities analyzed contribute to annual energy production and have the potential to reduce CO_2_ emissions. The differences in energy production and CO_2_ emission reductions between cities can be attributed to factors such as the availability of solar resources, system capacity, energy efficiency of buildings and the characteristics of the local grid. To further improve the analysis, it is important to collect additional data on the specific PV system configurations, including system capacity, efficiency, and maintenance practices. This would enable a more comprehensive assessment of the performance and environmental impact of rooftop PV systems.

### 4.5. Angular, spectral, temperature and low irradiance loss of the installed rooftop PV system

**Fig 9:**
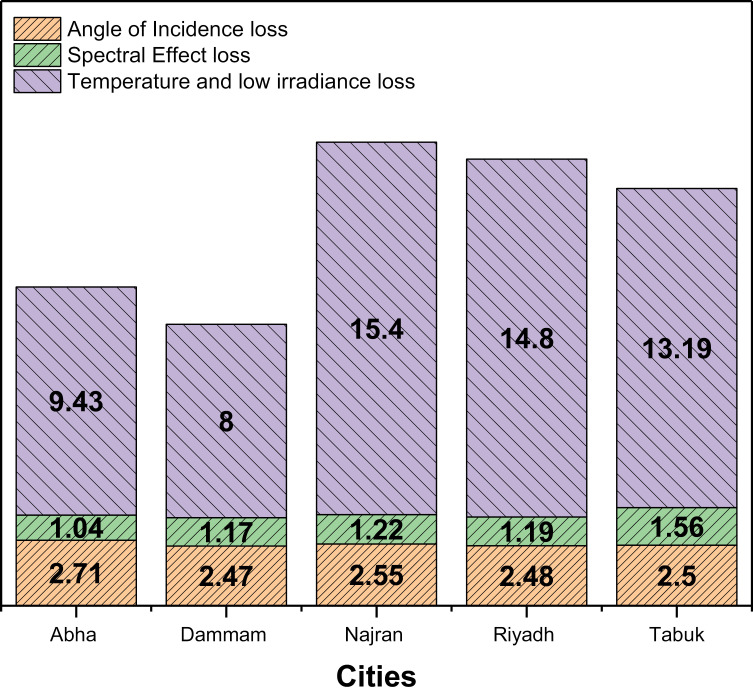
Angular, spectral, temperature and low irradiance loss of the installed rooftop PV system for different location.

Losses in efficiency in photovoltaic (PV) systems can significantly impact their overall performance. The factors in terms of losses that affect the performance of rooftop PV systems, such as angular, spectral, temperature and low radiation losses, are shown in Fig 9. Angle loss is the decrease in the efficiency of the PV system due to variations in the incident solar radiation angle [53,54]. The results show that all cities experience angle losses between 2.47% and 2.71%. The relatively low angular losses at all locations suggest that the installed rooftop PV systems are optimally oriented and effectively capture sunlight throughout the day [55,56]. However, further research is required to determine the specific design and alignment strategies used to achieve these low losses. Spectral effects occur when the performance of the PV system is affected by changes in the incident solar spectrum. The results show spectral losses between 0.84% and 1.36%. The different spectral losses between cities may be due to differences in atmospheric conditions, such as air pollution or cloud cover. In addition, the design of the PV system, including the choice of solar cell technology and anti-reflective coatings, may contribute to the observed spectral losses. Temperature and low irradiation losses are responsible for the decrease in PV system efficiency caused by high operating temperatures and reduced solar radiation. The results indicate temperature and low radiation losses ranging from 8% to 15.4%. The higher losses observed in Najran, Riyadh and Tabuk may be due to the hot climatic conditions leading to elevated operating temperatures. Proper thermal management techniques, such as ventilation or cooling systems, can help mitigate these losses. Low irradiance conditions, which may occur on cloudy days or in regions with less solar radiation, contribute to overall losses [57]. The higher percentage of low radiation losses in Najran suggests that periods of reduced sunlight availability are more common at this location. The results show that the installed rooftop PV systems in the analyzed cities generally have low angular and spectral losses. These results suggest efficient system design and proper orientation that enable optimal use of available sunlight. However, temperature and low radiation losses pose significant challenges for maintaining high system performance. The higher losses observed in certain cities highlight the need for effective thermal management strategies and careful consideration of local climatic conditions in system design [58,59]. To further improve the analysis, it is important to collect additional data on the specific PV system configurations, including the types of solar cells used, inverters, and any advanced technologies used to mitigate losses.

## 5. Conclusion

Analysis of the thermal and energy performance of rooftop photovoltaic (PV) systems in different climatic locations in saudi arabia has provided important insights into their operational efficiency and potential for energy production. The results show a direct correlation between climatic conditions and energy consumption patterns, with higher energy demand observed in the summer months due to increased reliance on cooling systems, particularly in hotter regions such as riyadh and dammam, where an average of around 25,000 kwh is used per year. in contrast, cooler areas such as abha have moderate consumption of around 19,000 kwh, highlighting the influence of local climate on energy demand. The study also indicates a bimodal energy demand pattern with peaks in both summer and winter, influenced by cooling degree days (CDD) and dry bulb temperature (DBT).

The study shows a strong relationship between annual energy production from rooftop photovoltaic (PV) systems and average global solar radiation, with output ranging between 49,810.29 kwh and 60,204.29 kwh in different cities. najran and tabuk have higher energy production due to favorable solar conditions, which is reflected in their average global irradiance ranging from 188.15 kwh/m² to 212.52 kwh/m². the evaluation of the annual energy production and co_2_ emission reduction from rooftop photovoltaic (PV) systems in various saudi cities shows their significant environmental benefits and energy production potential with outputs between 49,810.29 kwh and 60,204.29 kwh. Cities such as najran and tabuk show higher energy production and significant co_2_ reductions between 1,848.96 kg and 2,234.78 kg due to favorable solar conditions. These systems effectively meet the energy needs of buildings. The angular and spectral losses are relatively small - ranging from 2.47% to 2.71% for angular losses and 0.84% to 1.36% for spectral losses, temperature and low temperature irradiation losses pose significant challenges to overall efficiency, with losses ranging from 8% to 15.4% vary, especially in hotter regions such as najran, riyadh and tabuk. These results highlight the importance of effective thermal management strategies to optimize system performance in response to local climatic conditions.

Overall, this research highlights the potential of rooftop PV systems to meet residential energy needs and contribute to sustainability goals in Saudi Arabia. The differences in energy consumption and energy production in different climate locations highlight the need for local strategies in the planning and implementation of PV systems. To maximize the benefits of solar energy, further research is needed to examine system configurations, energy efficiency measures, and the impact of local climatic factors on PV performance. The study provides more accurate assessments and enable improvements in the design, operation and policy development of PV systems, sustainable energy strategies and the transition to a low-carbon future, and can help policymakers, energy planners and solar industry professionals make informed decisions about the deployment of PV roof systems in different climatic regions.

## Supporting information

S1 File Simulated data. (XLSX)

## Abbreviations

ACH: Air change rate
ASHRAE: American Society of heating, refrigerating and air-conditioning engineers
CDD: Cooling degree days
CE: Cooling energy
CLF: Solar cooling load factor
CLTD: Cooling load temperature difference
DBT: Dry bulb temperature
EPD: Equipment power density
GHI: Global horizontal irradiance
HDD: Heating degree days
ICC: International code council
IECC: International energy conservation code
KSA: Kingdom of Saudi Arabia
LPD: Light power density
SBC: Saudi Building Code
SC: Shading coefficient
SG: Single glazed
SHGC: Solar heat-gain coefficient
WWR: Window-to-wall ratio.
